# Co-designing, measuring, and optimizing innovations and solutions within complex adaptive health systems

**DOI:** 10.3389/frhs.2023.1154614

**Published:** 2023-03-31

**Authors:** Maria Alejandra Pinero de Plaza, Lalit Yadav, Alison Kitson

**Affiliations:** The Caring Futures Institute, College of Nursing and Health Sciences, Flinders University, Adelaide, SA, Australia

**Keywords:** complex systems, implementation, evaluation, knowledge translation, digital health, healthcare, transdisciplinary co-design

## Abstract

**Objective:**

To introduce, describe, and demonstrate the emergence and testing of an evaluation method that combines different logics for co-designing, measuring, and optimizing innovations and solutions within complex adaptive health systems.

**Method:**

We describe the development and preliminary testing of a framework to evaluate new ways of using and implementing knowledge (innovations) and technological solutions to solve problems *via* co-design methods and measurable approaches such as data science. The framework is called PROLIFERATE; it is initially located within the ecological logic: complexity science, by investigating the evolving and emergent properties of systems, but also embraces the mechanistic logic of implementation science (IS) (i.e., getting evidence-based interventions into practice); and the social logic, as the study of individuals, groups, and organizations. Integral to this logic mixture is measuring person-centered parameters (i.e., comprehension, emotional responses, barriers, motivations, and optimization strategies) concerning any evaluated matter across the micro, meso, and macro levels of systems. We embrace the principles of Nilsen's taxonomy to demonstrate its adaptability by comparing and encompassing the normalization process theory, the 2 × 2 conceptual map of influence on behaviors, and PROLIFERATE.

**Results:**

Snapshots of ongoing research in different healthcare settings within Australia are offered to demonstrate how PROLIFERATE can be used for co-designing innovations, tracking their optimization process, and evaluating their impacts. The exemplification involves the evaluation of Health2Go (the design and implementation of an innovative procedure: interdisciplinary learning within an allied health service—community-based) and RAPIDx_AI (an artificial intelligence randomized clinical trial being tested to improve the cardiac care of patients within emergency departments—tertiary care).

**Conclusion:**

PROLIFERATE is one of the first frameworks to combine ecological, mechanistic, and social logic models to co-design, track, and evaluate complex interventions while operationalizing an innovative complexity science approach: the knowledge translation complexity network model (KT-cnm). It adds a novel perspective to the importance of stakeholders’ agency in the system by considering their sociodemographic characteristics and experiences within different healthcare settings (e.g., procedural innovations such as “interdisciplinary learning” for Health2Go, and tech-enabled solutions such as RAPIDx_AI). Its structured facilitation processes engage stakeholders in dynamic and productive ways while measuring and optimizing innovation within the complexities of health systems.

## Background

Globally, health systems are under pressure due to increased healthcare utilization and its associated demands. Limited access to health services is rising because of the increasing number of people living with more than one chronic condition and non-communicable diseases (NCDs), such as heart disease, cancer, diabetes, and others (1–7). Apart from NCDs and the rising demands around caring for aging populations (4, 5), the existing COVID-19 pandemic has demonstrated that the ramifications of a single communicable disease have far-reaching effects in terms of accentuating preexisting inequalities in healthcare (1–3, 6, 8). Although, between 1990 and 2019, the Healthcare Access and Quality (HAQ) index increased overall (19.6 points, 95% uncertainty interval 17.9–21.3), particularly in the young age group, it also indicates that healthcare access and quality are lagging among those belonging to lower levels of social and economic development (i.e., those of working age (15–64 years) and post-working age (65+ years)) (9).

Addressing current healthcare access and quality trends and responding to demands around better health services for patients with multimorbidity or populations affected by communicable diseases requires the implementation of changes in the health system *via* innovations and or solutions; these concepts refer to using either new knowledge or discovering new ways of using existing knowledge (innovations) and technology (knowledge activated by some technological solution) to solve health problems; for example, developing vaccines, devices, and diagnostics, new drugs, as well as new techniques or processes for designing, engineering, or manufacturing health products, treatments, healthcare management approaches, software, policies, and services (8, 10, 11). However, the implementation of any of these innovations requires a good understanding of the health system and their impact within so that the innovations can be accepted and utilized (i.e., uptake) across time (i.e., sustainability) or modified to work according to changes (i.e., optimized) or de-implemented when necessary. To that end, according to the WHO, “*a health system is most simply described as being made up of component parts (e.g., stakeholders and organizations), and interactions (e.g., functions) that promote, restore and maintain health and that, taken together, form a unified whole*” (1, p. 3).

As COVID-19 demonstrates (1–3, 6, 8), implementing innovations within ever-changing health system environments requires collecting and interpreting data about the *whole health system structure*, *functions*, *and parts*; this is done by gathering large datasets analyzed with computational methods (a procedure referred to as big data) (12, 13). Such approaches—collecting data about engaging people, their connection with each other and their environment (organizations), and their respective views—represent, for many businesses and technological sectors, a fundamental condition for assessing the impact of new knowledge in the shape of innovation (12–15). In this context, a crucial step for delivering person-centered healthcare services, policy, research projects, and programs is evaluating data with the contribution of such stakeholders (people affected by the innovation being implemented) (14, 15). Yet, working with stakeholders and their data involves a paradigm shift. For example, the transition requires, among other changes, moving away from a single disease model toward a more holistic one, in which the totality of the personal experience can provide essential insight about a solution relevant to the person and the health system; this refers to the evaluation of the effect and impact that the introduced solution may have, could have, or is having in light of its users’ feedback (9, 16, 17).

A lack of engagement with relevant stakeholders or their exclusion from analyzing information concerning their personal experience may bring negative consequences. For example, despite an overall increase in evidence-based resource allocation over the last decade, the distribution of such resources has mainly been determined by population-based surveys on risk factors and the existing scope of health and medical research, which is focused on disease- or discipline-specific outcomes (9, 16). A more holistic view of the health system can come from working with all groups affected by the change or innovation we are planning to introduce; yet, a clear gap in knowledge and practice has been identified concerning such attempts (1–3, 8, 18, 19). For example, the understanding and utilization of co-design methods for research, implementation, and evaluation purposes is often referred to as poor when co-design is understood as the meaningful involvement of users of innovation or solution across all discovery phases (1–3, 8, 18, 19). In this way, there is an increasing demand for shepherding the data collection and analysis of big data to guarantee that their interpretation reflects the issues of relevance for patients, families, communities, and other stakeholders. This is still an issue for decision-makers, academics, and practitioners as more data are increasingly collected and analyzed from a limited or partial perspective that needs to reflect the dynamics of the health system, its functions, and its parts (1–3, 8, 9, 16, 18, 19).

In the context of co-design, meaningful involvement means the non-tokenistic participation of stakeholders. Their roles and contributions must be explicitly described, defined, and auditable across all processes of a project; this means that their tasks and functions confer them influential voices and decision-power (14, 15, 19). This type of democratization of research, implementation, and development processes permit the capturing of important insights on the attributes and connections between people and groups and eventually incentivize change (i.e., better knowledge uptake and health) (1–3, 8, 18, 19). For example, today, the investigation of the health system through the lens of people’s experiences, may capture information about climate change and war. These subjects may seem separate from implementing a health solution. Still, those events cause injuries and illnesses within many populations and disrupt the operation of healthcare facilities and health due to migration, poverty, food insecurity, racism, and other socioeconomic issues (1–3, 8, 18, 19). These connections between topics and sectors that initially seem disconnected from implementing health innovations demonstrate that implementing change within health systems extends beyond the health sector. Therefore, implementing changes for better delivery and access to healthcare services broadens to considering the social determinants of health (e.g., biological, socioeconomic, political, and psychological factors affecting individuals and the promotion or restoration of their health) (1).

The poor engagement of stakeholders from different sectors may limit the ability of researchers and decision-makers to capture important information and the capacity to interpret people’s experiences concerning innovation (14, 15, 19). For instance, when implementing and evaluating a new treatment, failing to engage stakeholders can limit the researchers’ interpretation of data patterns on the behavior of organizations; e.g., hospitals and communities, and their functions (e.g., healthcare services and their utilization) concerning the treatment's positive and negative implications according to its different users (e.g., clinicians delivering the treatment) or other end-users (e.g., patients, family members, and their reported changes on their roles and behaviors as a result of the treatment implementation).

A systematic review and meta-analysis of consumer engagement in healthcare policy, research, and services has come to the same conclusions as studies addressing longitudinal solutions around urban development and community health (14, 15). Both studies recommend using participatory methods such as co-design and quantifiable approaches (e.g., big data). Their commonality appreciates the importance of measuring engagement for the sustainability of the implementation of innovations and their uptake; this refers to the scale-up or the adoption of the behaviors that the innovation requires from its different users (14, 15, 19). These strategies are relevant to facilitate a better distribution of power and expertise throughout the discovery, implementation, and evaluation processes because they result in innovations informed by insights from experts and the knowledge gained through people's lived experiences across all relevant sectors involved (14, 15, 19).

### Research objective

The presented background concerning healthcare utilization, innovation, co-design, and its relevance for implementing and influencing change within the health system coincides with other studies that suggest tapping into different logics or methods to achieve better implementation uptake and health (5, 20–22). Those studies imply that implementation and its uptake within health systems is not only about bringing evidence-based interventions into practice (known as mechanistic logic). It requires considering the evolving and emergent properties of the person's networks (identified as the ecological logic) and the study of the social organizations, groups, and individuals (covered by the social logic) (20, 22). Mixing or integrating these logics and their most relevant methods could help to recognize effective implementation processes for innovations and the best approaches for their sustainability and optimization (or not) considering their relevant contexts and settings within the health system (5, 20–22):

1. *Ecological logic*: complexity science, the field investigating the evolving and emergent properties of systems (15).
2. *Social logic*: social science, which concentrates on the social study of individuals, groups, and organizations (15).
3. *Mechanistic logic*: implementation science as the field bringing evidence-based interventions into practice (15).

As introduced in the background, a practical combination of ecological, mechanistic, and social logic is required, focusing on co-design and measurable approaches. This need is reflected in reviews demonstrating that millions of dollars are lost yearly in implementing health innovations that still need to achieve their expected uptake despite being backed up by robust evidence of their benefits (19, 20, 22). Consequently, responding to the need for such a methodological integration, this manuscript introduces, describes, and demonstrates the emergence and testing of an evaluation method that combines those logics for co-designing, measuring, and optimizing (or not) innovations and solutions within complex adaptive health systems.

## Methodology

Initially, the task of combining different logics was triggered by evaluating the impact of a video on stakeholders’ perceptions of frailty, described elsewhere (23). The video was co-designed with health consumers, e.g., patients and their carers, health researchers, and clinicians (this aspect of the video is consistent with social logic objectives). However, the video was created to disseminate and facilitate the utilization of evidence-based information for managing frailty (objective aligned with mechanistic logic) by reflecting on consumers’ experiences and priorities (which also reflects the characteristics of the ecological logic) (23). Evaluating this combination of aspects from the perspectives of the video's stakeholders (i.e., users such as health promoters and partitioners and end-users such as patients) was essential to understanding and measuring its impact. Therefore, a group was created to integrate a practical way of evaluating the video. The group involved many stakeholders who were not involved in the video creation team (23) but with the representation of different sectors (areas of knowledge) associated with the audiovisual resource:

• clinicians from different disciplines;
• artists;
• a mass communicator;
• health researchers; and
• health consumer advocates.

**Figure 1:**
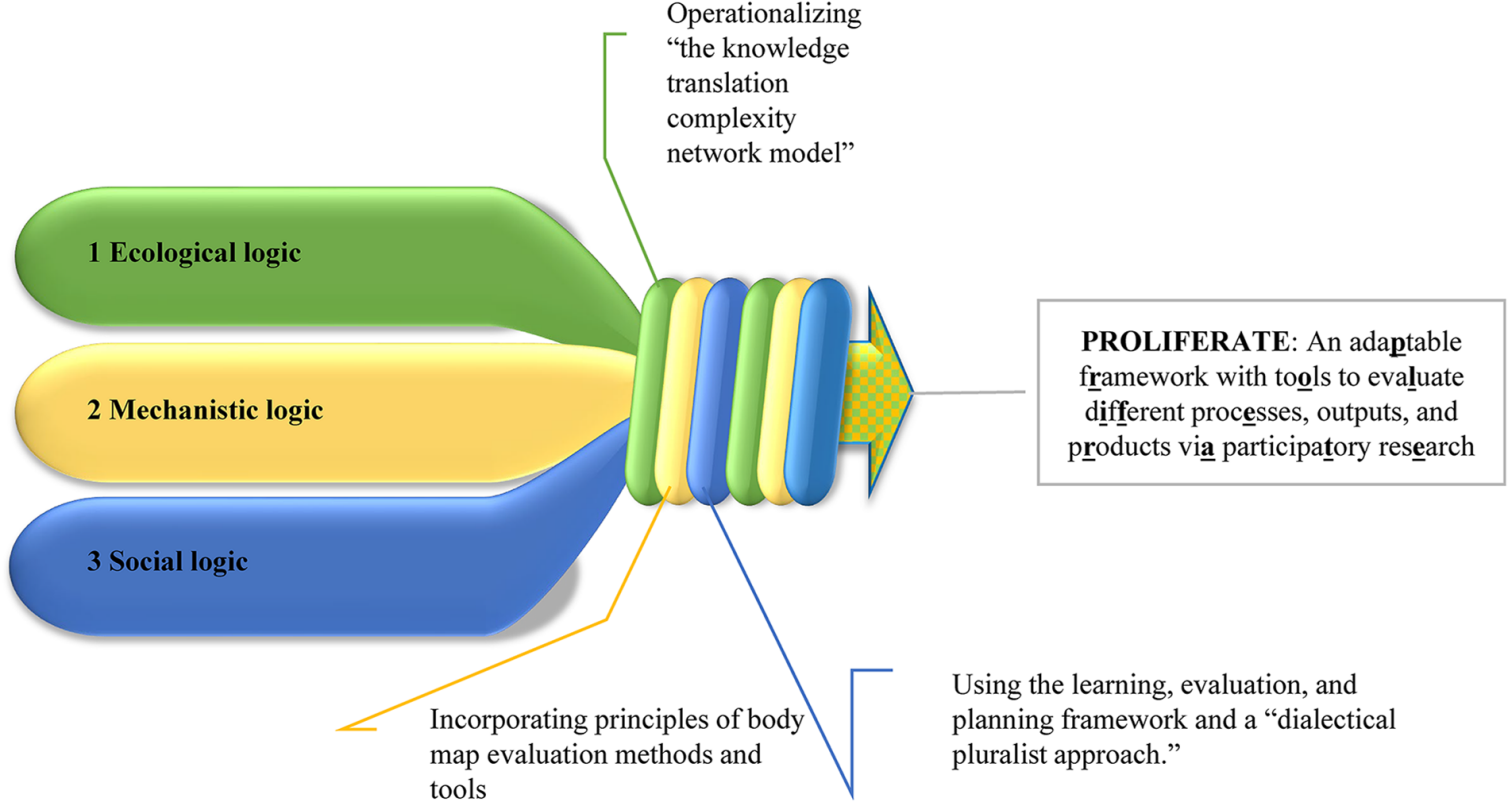
Development of the PROLIFERATE framework. The transdisciplinary group has been working together for 3 years, on which COVID-19 changed the operative dynamics toward online communications and meetings. Benefitting mostly from iterative online discussions and presentations to peers, health consumer advocates, and health practitioners, the group inductively integrated the combination of logic presented in Figure 1 and Table 2 (24, 28–30). The mixture of their personal and methodological experiences, skills, reasoning, and areas of research resulted in a high-level combination of procedures and methods to evaluate different processes, outputs, and products *via* participatory research: PROLIFERATE. It is required to gain familiarity with the evaluation methods, mixed-methods research approaches for achieving spread, and scale-up of innovation explained in Tables 1 and 2 and illustrated across all figures of this manuscript to comprehend the PROLIFERATE framework, its focus, procedure, and its operationalization.

This group of stakeholders decided to work using a transdisciplinary approach; this means incorporating the knowledge from their different disciplines and experiences to produce an evaluation that transcends the boundaries of their various fields. This transcendence aspect means moving their contributions to areas beyond their personal experiences/disciplines to create, together, a new way of evaluating the impact of the video, considering its different aspects/logic (24–27). The resulting video evaluation procedure is summarized in Figure 1 and explained in Table 1.

**Table 1:** The combination of logic that integrates the introduced evaluation framework (table adapted from Greenhalgh and Papoutsi, 2019) (22).

Logics	Focus	Contribution	Core processes of spread and scale-up of innovation	Key methods for achieving spread and scale-up of innovation	Key methods for researching spread and scale-up of innovation	Measures and metrics	Evaluation approaches combined to create PROLIFERATE
*Ecological*: Complexity science	The evolving and emergent properties of systems	Emphasizes the system's inherent complexity and need for adaptive change at multiple interacting levels	Emergent properties of an interacting system—self-organization, management of interdependencies, and sense-making	Achieve a rich understanding of the case context. Use multiple methods flexibly and adaptively. Assumes surprises and manages them inventively. Builds on individuals and organizations to be creative and resilient	Case study approach using multiple qualitative and quantitative methods. Narrative can be used as a synthesizing tool to capture complex chains of relationships and structures	Conjugated narrative about what changed and why including (where relevant) how the intervention was adapted or abandoned	The knowledge translation complexity network model (KT-cnm)—explained in Table 2 and Figure 3—whose objective is to initiate new ways of thinking, engaging with complex problems and generating relevant solutions that can become the accepted standard (31). It helps to identify users/adopters represented in the five sectors (Governments; Community, including Industry; Health; Education; and Research) that function dynamically in space and time as networks/clusters that differ in frequency of interaction and goals across the KT stages/processes (Problem identification, Knowledge creation, Knowledge synthesis, Implementation, and Evaluation) (31).
*Mechanistic*: Implementation science	Evidence-based interventions in practice	Provides concrete and planned approaches to the delivery and study of spread and scale-up	Uncertainty reduction, emphasis on fidelity, and contextual influences	Use structured, programmatic approaches to develop and replicate a complex intervention across multiple settings	Metrics for measuring improvement (quantitatively) and systematic approach to exploring processes and mechanisms (qualitatively)	Replication of a particular service model or approach in multiple contexts (“fidelity”)	Body map evaluation tools—illustrated in Figure 4—which tend to be used to investigate and measure people's perceptions, opinions, beliefs, and attitudes, by capturing evidence from those with limited literacy or language differences, involving people while exploring complex processes, capturing many opinions and views efficiently; gathering impressions of progress or outcomes and providing evidence of unexpected outcomes (32, 33)
*Social*: Social science	Social study of organizations, groups, and individuals	Focuses on patterns of social behavior and interaction, professional beliefs and values, and organizational routines and structures	Social, professional, and organizational influences that shape (and are influenced by) individual and collective action	Develop and apply theories of how individuals’ behaviors and actions are influenced by interpersonal, material, organizational, professional, and other factors	Ethnography, interview-based methods, and case narratives to provide insights into social interactions and contexts	Theoretically informed and empirically justified explanations about human and organizational behavior	The learning, evaluation, and planning framework (LEAP) (34), which is a resource used by those involved in promoting health and well-being in community settings, whether in community projects, primary care, clinical practice, health promotion, or public health. It is designed to assist projects and programs that focus on enabling people to develop and maintain their health on a day-to-day basis through individual and participatory community action research (35). This method was combined with *a dialectical pluralist approach*, which refers to the acceptance and expectancy of differences in every aspect of the evaluation by facing them with a dialogical attitude thriving on differences and intellectual tensions (36)

**Figure 2:**
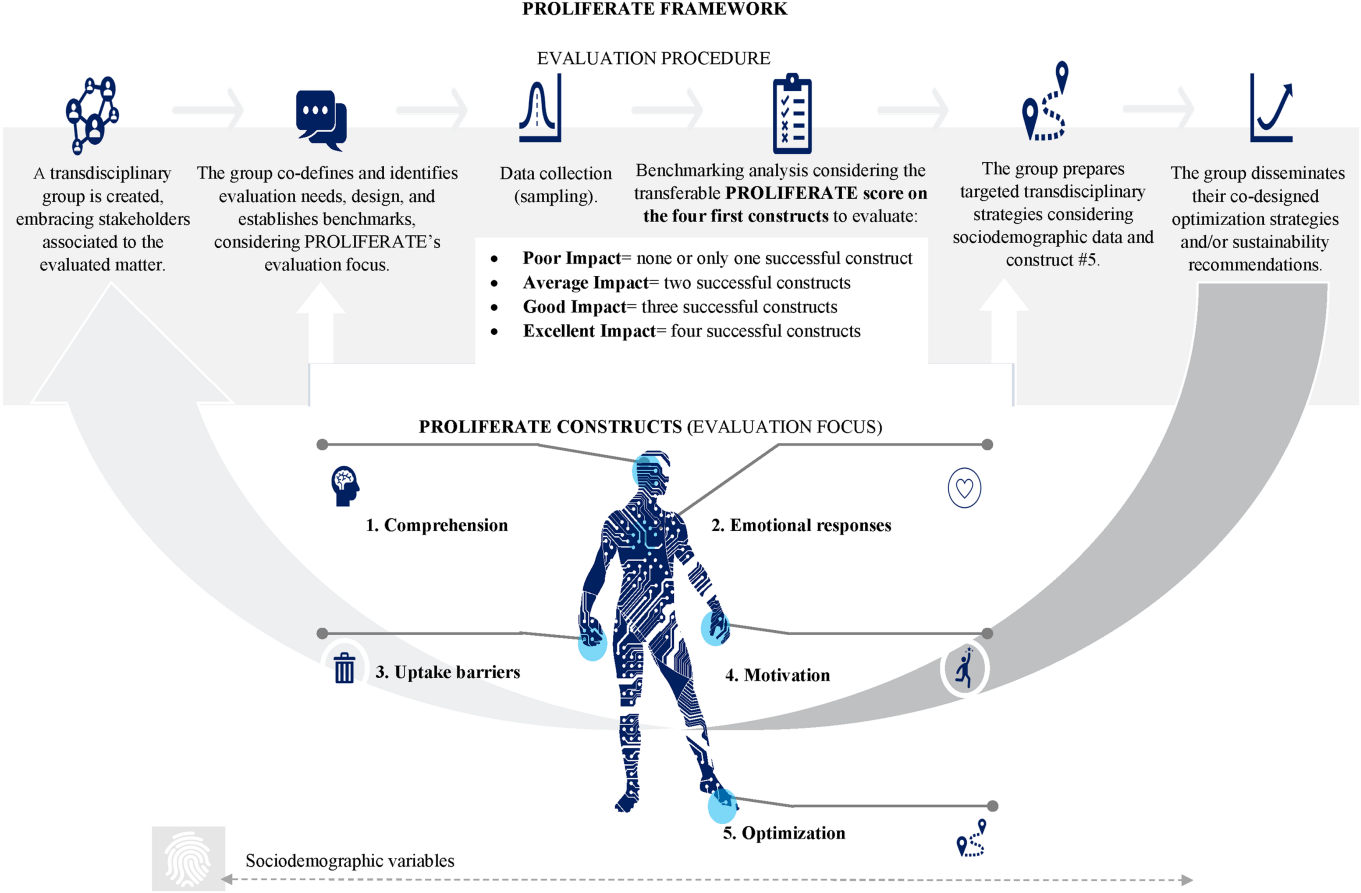
PROLIFERATE framework (evaluation procedure and focus components) (24, 28–30).

The multi-logic combination of Table 1 was eventually adapted, modified, and applied to other projects evaluating different innovations and involving their respective transdisciplinary groups. These iterations resulted in a combination of the methods reflected in Figure 2 and used in the exemplar cases presented in the “Results” section of this manuscript (24, 28–30).

### PROLIFERATE ecological logic: the basis of the evaluation

Comprehending Table 1 and Figure 2 involves understanding the knowledge translation complexity network model (KT-cnm), which is defined as a network that optimizes the effective, appropriate, and timely creation and movement of knowledge to those who need it to improve what they do (31). The model is a core component of PROLIFERATE because, by operationalizing it, evaluators can identify ways to identify the movement, adaptation, and acceptance of innovations within complex and adaptable processes; this is because they are dependent upon the decisions and actions of individuals and teams, and their connections across and between multiple networks (31). The KT-cnm is based on the concepts and definitions presented in Table 2 (replicated with authorization (31)).

**Table 2:** The nomenclature used and working definitions of the knowledge transition (KT) complexity network elements (31).

Term	Explanation
Node	A single agent (individual, process, or virtual system) that interacts with other single agents (nodes)
Hub	A single agent that interacts more extensively with other nodes and becomes the champion for collective actions, within and between clusters
Cluster	A subnetwork made up of nodes and hubs. The sub-network comprises a number of nodes, some of which act as hubs, pursuing the same goals
	A cluster may be a subnetwork involved with key areas of activity (such as PI) or a subnetwork within a sector (such as a university health science research group)
Network	A collection of nodes, hubs, clusters, and the connections between them
Problem identification (PI)	The process by which societal challenges, issues, or problems are formulated, defined, and constructed to proceed to systematic investigation
Knowledge creation (KC)	Describes what is traditionally termed basic, clinical, pre-clinical, epidemiological, health services, and population health research approaches to answering health related problems
Knowledge synthesis (KS)	The rigorous and systematic generation of evidence-based products (patents, materials, tools, programs, and guidelines) for application in policy and practice
Implementation (I)	The rigorous application of new knowledge into policy and practice in a theory informed and reflective way
Evaluation (E)	The explicit and systematic review of key processes of KT and broader objectives within and across a range of complex and interconnected sectors and networks
Complex adaptive system (CAS)	Complex systems (e.g., within research institutions, health systems) and KT processes (e.g., PI, KC) that are a collection of diverse connected nodes or parts with interdependent actions. The behavior of a CAS is generated by the adaptive interactions of its components
KT complexity network	The umbrella term that describes the components of the overall network that connect and interplay in order for KT to occur. Different stakeholders collaborate within a dynamic discursive space to ensure that appropriate information is being developed, refined, and mobilized throughout the network to the appropriate nodes, hubs, clusters and sectors

**Figure 3:**
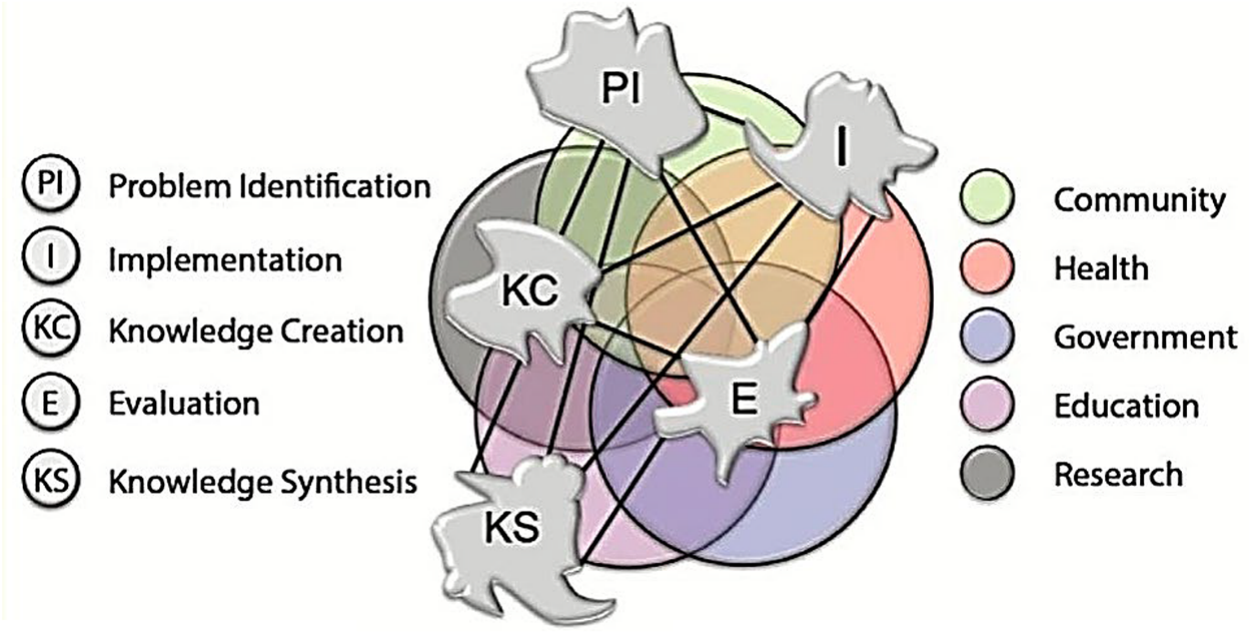
The knowledge translation complexity network model (KT-cnm) (31). The overlapping of lines (connection between KT stages) and colors (different sectors) represent the complexities of the ever-changing conditions of a complex adaptive system (CAS).

The KT-cnm (31) brings the ecologic basis of PROLIFERATE (defined in Table 1 and illustrated in Figure 3). Having the KT-cnm as a foundational component of PROLIFERATE is of interest to the field of IS because it means operationalizing a novel way of considering the evolving and emergent properties of the health systems and developing, identifying, implementing, and evaluating solutions that attempt to respond to the challenges presented in the introduction (22, 31, 37).

PROLIFERATE was designed to help to identify relevant stakeholders (nodes, hubs, clusters, and networks) represented in the five sectors of Figure 3 (Governments; Community including Industry; Health, Education, and Research) and aims to view and measure how those sectors function dynamically in space and time as clusters that differ in frequency of interaction and goals across the KT stages/processes (Problem identification, Knowledge creation, Knowledge synthesis, Implementation, and Evaluation) (see Figure 3 and explanations in Table 1) (31).

The measuring objectives of the PROLIFERATE evaluation framework given the KT-cnm also take a mechanistic logic (as per Table 1) and therefore are compatible with IS broader methodological objectives because PROLIFERATE aims to (21):

1. describe/guide the process of translating research into practice *via* process models (21);
2. understand/explain what influences implementation *via* determinant frameworks, classic theories, and implementation theories (21); and
3. evaluate implementation *via* evaluation frameworks (21).

**Figure 4:**
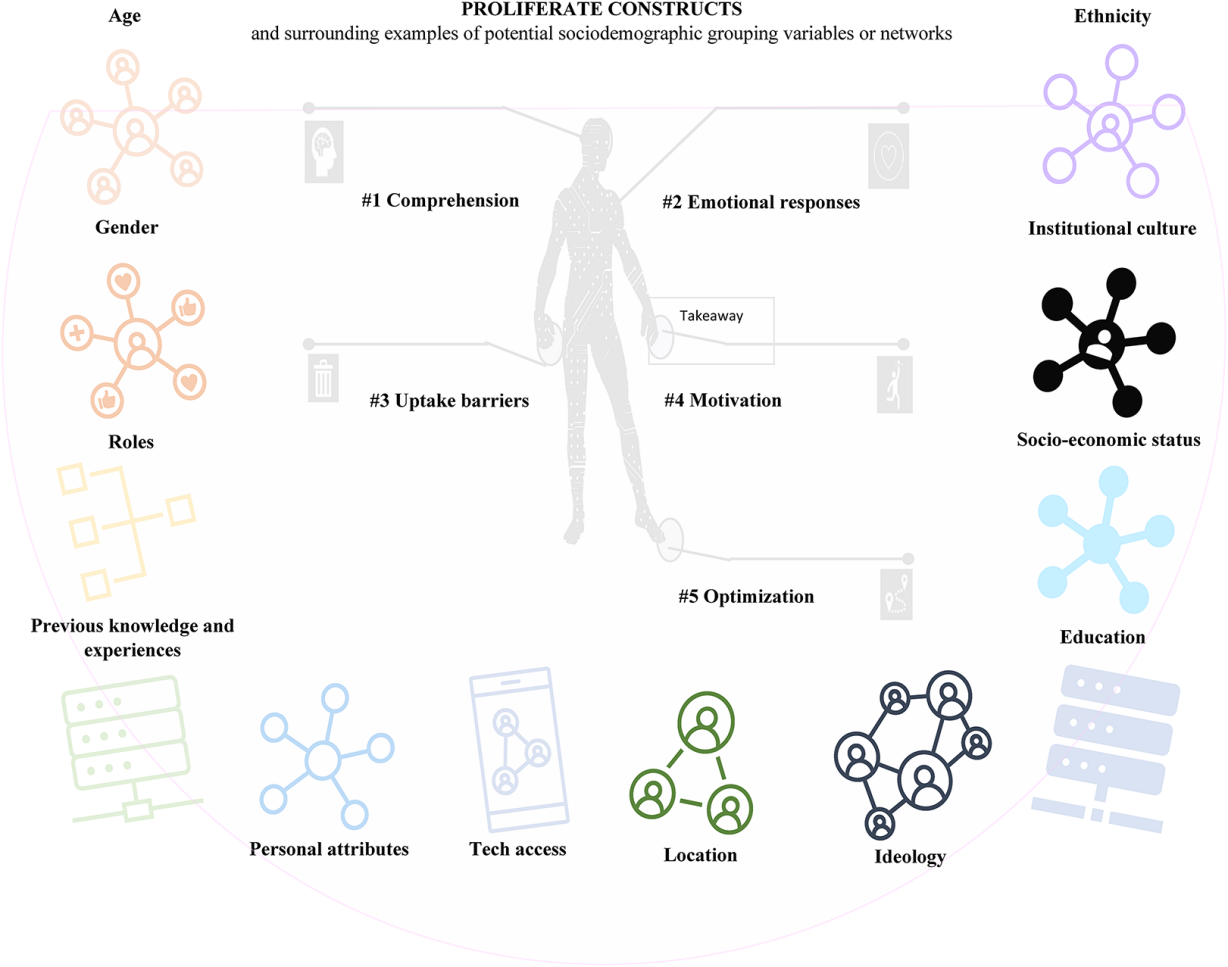
PROLIFERATE's five non-hierarchical constructs and the person's sociodemographic characteristics (i.e., examples of variables for creating possible affiliation networks—grouping—to map multilevel social networks).

Research into IS methods and practice indicates that methods combining the above objectives tend to be too general (lacking details in their how-to components) or too specific, needing more transferability (21). Therefore, the PROLIFERATE design tries to bring balance between such extremes (i.e., becoming too general or too specific) by using the principles of body map evaluation tools (see Table 1) (32, 33), to establish constructs (i.e., person-centered parameters) that can capture the stakeholders’ comprehension, emotional responses, barriers, motivations, and optimization strategies concerning any evaluated matter (32, 33), while evaluating stakeholders’ sociodemographic characteristics to assess their interactions with their context and grouping them (see Figures 2 and 4).

### PROLIFERATE mechanistic logic: the focus of the evaluation

Figure 4 provides a simplified view of the five non-hierarchical person-centered parameters of PROLIFERATE called constructs; they were included in the PROLIFERATE design as the focus of the evaluation (as per Figure 2) to explore the person construction of meaning or their perceptions concerning any evaluated matter. Evaluating these constructs in combination with the person's sociodemographic characteristics (or variables) is essential, as such variables play a similar role to the social determinants of health mentioned in the background of this manuscript (1). To that end, the constructs can help to capture and measure the multiple interacting social structures and ecological networks by identifying the stakeholders’ functions within the KT-cnm sectors of the health system.

Ideas around the constructs’ measurement and their mapping *via* network structures (grouping) were imported by members of the PROLIFERATE transdisciplinary group and their in-depth explorations of fundamental care (31, 38, 39). Their work quantifies and maps a network structure considering the micro, meso, and macro dimensions of care as explained elsewhere (38, 39). The parallelism between PROLIFERATE and such measurement of fundamental care is based on the analysis of networking data about the personal experience of patients, clinicians, and care administrators, to eventually develop interventions through a thorough investigation of the intersections of 38 fundamental care elements, which are similar to the five person-centered constructs in the context of the individual sociodemographic characteristics as variables (Figure 4). The combination of constructs and variables in the described PROLIFERATE evaluation focus (presented in Figure 4) unites the ecological and social logics of Table 1 to capture and measure (*via* a mechanistic logic) different stakeholders’ views/perceptions and their networking position and functions within the health system—they can be policymakers, implementers, community members, managers, providers, and other types of innovation users/roles (1, 31, 38–41).

The non-hierarchical focus that PROLIFERATE constructs have allows for the creation of a simple and transferable scoring system, which permits to evaluate and track the stakeholders “software”: the agency that shapes human behavior, ideas, interests, values, norms, and/or the conscious and unconscious drivers impacting on innovation spread and scale-up, e.g., by incorporating experiments about human perception and behavioral responses around the constructs concerning an innovation (1, 42). This scoring system is designed to analyze results according to the pre-established benchmarks (quantifiable measures of success) that the transdisciplinary group sets for each construct at the beginning of the evaluation (see in Figure 2, the PROLIFERATE procedure, and the evaluation focus being united by the scoring system).

The score introduced in Figure 2 helps the transdisciplinary group determine conclusions about the quality, merit, or worth of the evaluated matter irrespective of the analytical methods utilized to benchmark the success of each of the four first constructs of PROLIFERATE (e.g., qualitative, quantitative, and mixed methods). The score identifies the innovation impact as follows:

• *poor impact*: none or only one of the first four constructs presents data above the pre-established benchmark on success (hypothesized responses/behaviors toward innovation uptake, spread, and scale-up);
• *average impact*: two of the first four constructs resulted in data going above their pre-established benchmark;
• *good impact*: three of the first four constructs resulted in data going above their pre-established benchmark; and
• *excellent impact*: the first four constructs resulted in data going above their pre-established benchmark.

The framework's fifth construct (#5) refers to “optimization” and is designed to capture the person's qualitative feedback on improving or modifying the evaluated innovation. This design facilitates a more meaningful interpretation of the score. The transdisciplinary group uses the score to determine if the implementation of the innovation needs to utilize optimization strategies to move the data obtained about the constructs above the pre-established benchmark (e.g., behavioral interventions, education *via* communication or campaigning, using creative, artistic, or empowerment activities, utilizing facilitation techniques, generating health interventions, re-design or re-engineering technology, etc.). For example, work on optimization strategies is required when the score results in poor, average, or good impact. If the score reflects excellent impact, sustainability strategies are necessary (i.e., activities that facilitate the maintenance of the status quo), as well as their monitoring across time.

**Figure 5:**
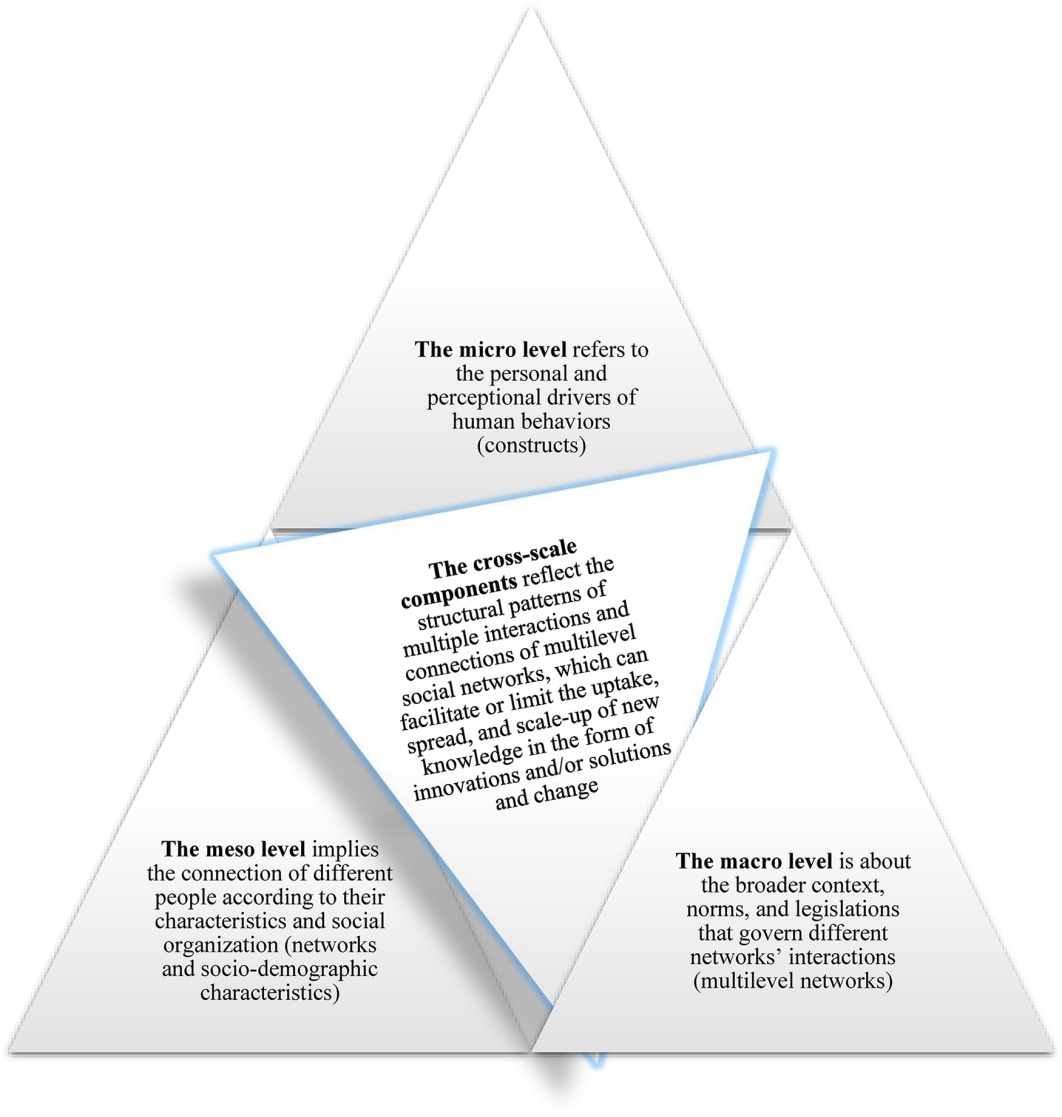
PROLIFERATE multi-logic is centered on the cross-scale components of health systems.

Construct #5 can also be used (in combination with the sociodemographic variables) to assess the de-implementation of an innovation or a solution; to this end, the standardization of assessments that the score provides could help compare different evaluated matters across their sectors/users. Using the described network approach and benchmarking stakeholder feedback concerning the constructs and the person's sociodemographic variables can help to navigate across these levels of health systems by exploring their dynamics, i.e., cross-scale components (as presented in Figure 5) (1, 31, 38–41):

• The micro level refers to the personal and perceptional drivers of human behaviors (the five person-centered constructs).
• The meso level implies the connection of different people according to their characteristics and social organization (networks and their sociodemographic characteristics).
• The cross-scale components reflect the structural patterns of multiple interactions and connections of multilevel social networks, which can facilitate or limit the uptake, spread, and scale-up of new knowledge in the form of innovations and/or solutions and change.
• The macro level is about the broader context, norms, and legislations that govern different networks’ interactions (multilevel networks).

**Figure 6:**
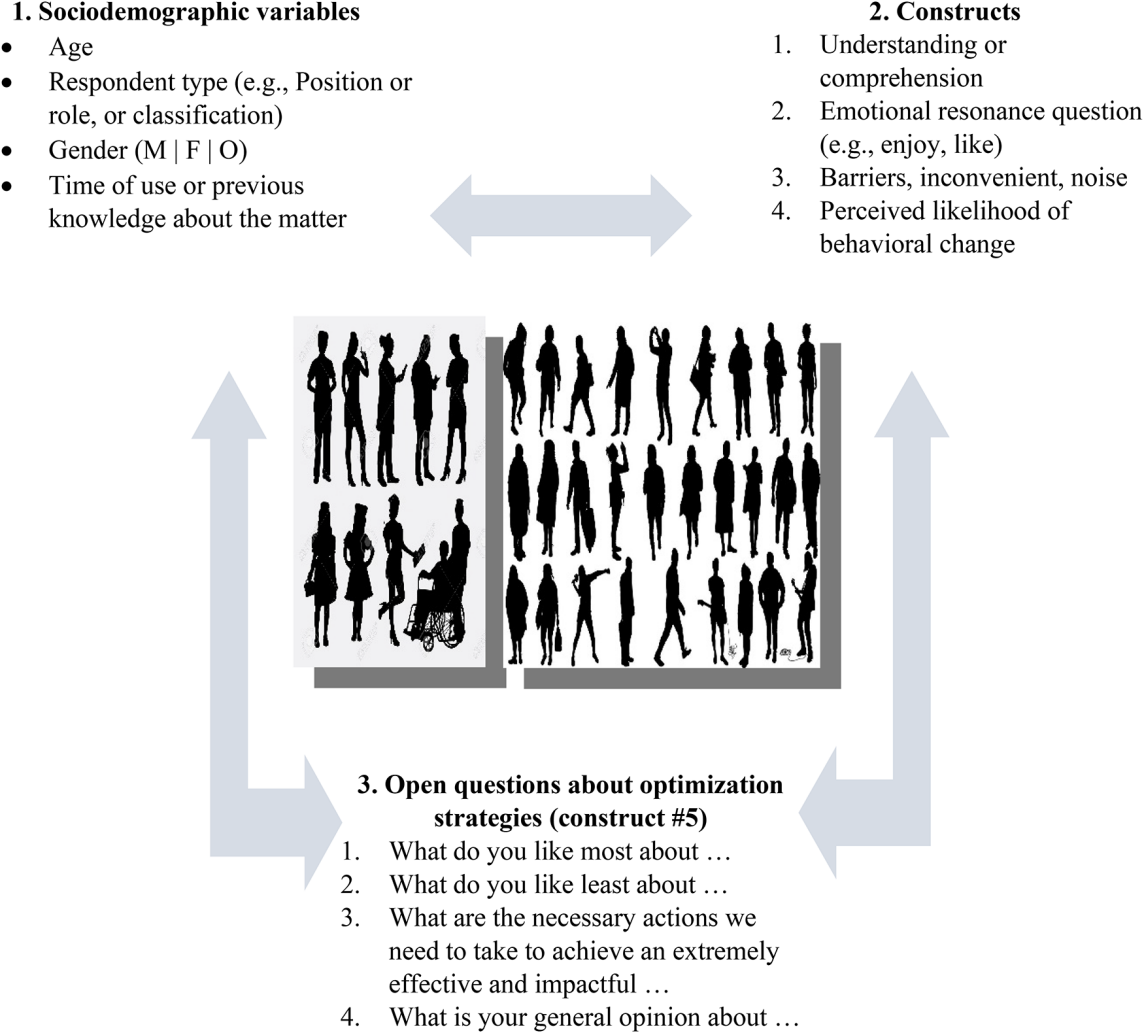
An example of survey items/questions for sampling about cross-scale components *via* PROLIFERATE (28).

Figure 5 implies that the PROLIFERATE constructs and the person's sociodemographic characteristics (possible grouping variables of Figure 4) provide information about the system, contexts, and settings from which different stakeholders perceived any innovation (26, 33–36). To illustrate how the PROLIFERATE cross-scale components can be considered/captured *via* sampling procedures (e.g., survey), Figure 6 (28) shows a general view of the triangulation structure between possible survey items or questions concerning the following:

1. sociodemographic variables;
2. constructs #1, #2, #3, and #4; and
3. potential open questions around optimization (construct #5, to be utilized and triangulated with and by the insight of the transdisciplinary group).

PROLIFERATE can be used longitudinally or cross-sectionally; this evaluation focus is implied in the bottom part of Figure 2, which shows an arrow that connects the last step of the procedure to its beginning, represents an iterative optimization cycle that improves and maintains an ongoing application of the framework if necessary. This quality and sustainability cycle assumes the implementation and de-implementation of interventions, procedures, and technologies as a natural process that requires measuring the tracking/scoring/benchmarking of the four first constructs and optimizing innovations (considering construct #5) within complex adaptive systems. A description or mapping of the cross-scale components can be done by examining the structural patterns, frequencies of interactions/networks, or structures of social relations and/or their types of connections and occurrence (17, 31, 38, 39).

### PROLIFERATE social logic: guiding procedure of the evaluation

The explained focus of the PROLIFERATE evaluation is guided by a procedure imported from the social logic of “the learning, evaluation, and planning framework” (LEAP) (34) (see Table 1 and the top part of Figure 2). The LEAP uses a co-design approach that steers the evaluation of PROLIFERATE constructs according to this general checklist (depicted in the top part of Figure 2):

• forming a transdisciplinary group with users of the evaluated matter and learning about the PROLIFERATE evaluation method;
• agreeing on the outcomes to be evaluated: goals and assumptions of the group, including the populations that they may be representing (e.g., KT-cnm sectors) and from which they will collect data to test their hypotheses; this means benchmarking (i.e., establishing measurable success indicators) concerning PROLIFERATE's constructs;
• actioning a planning process on making a difference about the most appropriate methods for collecting (sampling) and analyzing data about the innovation/solution, considering the constructs and the stakeholders’ sociodemographic characteristics as variables (as per Figures 3 and 6);
• tracking constructs to measure the innovation impact longitudinally or cross-sectionally; this means evaluating the difference made using PROLIFERATE scoring system and considering the optimization data (construct #5);
• creating targeted strategies for better uptake or optimization, considering each type of user (e.g., their KT-cnm sectors and their positioning according to the KT-cnm's stages (31)); and
• disseminating the lessons learned to each stakeholder group/type (i.e., targeted strategies developed from the data analysis) to facilitate the scale-up and sustainability of the evaluated matter (34, 35).

The whole procedure of PROLIFERATE is based on and supported by two enabling values from the social logic (36):

1. value 1: “pluralism” as the acceptance and expectancy of difference in transdisciplinary co-design environments (36); and
2. value 2: “dialectical” is the operative process of dialogical nature in which all positions have a voice and vote in the co-design table (36).

This “dialectical pluralism” component of PROLIFERATE is visible in Figure 2 (procedure). It is highlighted because of the need for proper co-design in the innovation process and the interpretation and evaluation of data and big data per stakeholders’ requirements. Therefore, all stakeholders, including the facilitators and evaluators, must work as peers with equitable expertise and authority to run this procedure. This enabling factor may allow researchers, practitioners, clients, policymakers, the community, and other stakeholders to co-design, track, influence, or optimize sustainable innovations or adaptable solutions accounting for their agreements, frictions, compressions, and tensions (36, 43, 44).

### PROLIFERATE as an adaptable evaluation framework

In making a case around the multi-logic of PROLIFERATE and demonstrating why it expands the body of knowledge to inform IS, we embrace the principles of Nilsen's taxonomy (around the characteristics of IS frameworks) (21). This is done by tabling a broad comparison of different IS approaches (Table 3). This comparison helps the reader identify *what* is required and/or *how* each method reaches its objectives. Table 4 facilitates a testing or comparison exercise intending to demonstrate the complementary nature between the different logics, approaches, designs, and capabilities of:

1. the normalization process theory (NPT) (45, 46);
2. the 2 × 2 conceptual map of influence on behaviors (42); and
3. PROLIFERATE (24, 30).

**Table 3:** Comparison of approaches (based on the principles of Nilsen’s taxonomy around the characteristics of IS frameworks (21)).

Approaches	Objectives	What is the investigative focus of the implementation approach	How users/adopters (e.g., clinicians) are addressed in the approach	How end-users are investigated or considered (e.g., patients) in the approach	How is the implementation context considered in the approach	How or what are the means of facilitating the implementation process
Normalization process theory (NPT) (45, 46)	Describing how the intervention and its components change and how practices are operationalized, enacted, and reproduced (Intervention performance, Relational restructuring, Normative restructuring, Sustainment –normalization)	Constructs (Coherence-building, Cognitive participation, Collective action, Reflexive monitoring) and mechanisms of purposive social action behind health techniques, technologies, and other complex interventions	Detecting the mechanisms of purposive social action that require an investment of personal and group resources	They are interpreted chiefly from the users’/adopters’ (e.g., clinicians) feedback	Qualifying patterns of social relations and structures (Strategic intentions, Adaptive execution, Negotiating capacity, Reframing organizational logic) that unfold over time and across settings and create the implementation environment	Identifying, characterizing, and explaining the Context-Mechanism-Outcome configuration to qualify and organize inductive and/or deductive implementation studies
2 × 2 conceptual map of influence on behaviors (42)	Guiding about the type of theory, model, or framework that might be most relevant for understanding and facilitating behavior change	Individual-level and collective-level influences on behaviors directed by conscious cognitive processes or non-conscious processing	Identifying the conditions regarding what influences practitioners’ behaviors	They are interpreted chiefly from the users’/adopters’ (e.g., clinicians) feedback	Using mixed-method research that responds to the types of influences on the behaviors that need to be changed	Accounting for whether behaviors are deliberate or automatically performed and under what circumstances they operate
PROLIFERATE (24, 30)	Co-designing, measuring, and optimizing innovations and solutions within complex adaptive health systems	Constructs around stakeholders, users, and end-users behaviors (Comprehension or understanding, Resonance or Emotional responses, Uptake barriers, Motivation associated with behavior change, and Optimization suggestions or opinions), and the person's sociodemographic characteristics as reflections of KT-cnm's networks/clusters/dynamics (i.e., PROLIFERATE: cross-scale components)	Co-designing: identifying, engaging, and sampling/testing/falsifying the dynamic interactions (in space and time) of the sample and their personal experience/expertise concerning their constructs, networks/clusters per sociodemographic characteristics across the KT-cnm processes/sectors and goals (i.e., PROLIFERATE: cross-scale components and insights from the transdisciplinary group)	Co-designing: identifying, engaging, and sampling/testing/falsifying the dynamic interactions (in space and time) of the sample and their personal experience/expertise concerning their constructs, networks/clusters per sociodemographic characteristics across the KT-cnm processes/sectors and goals (i.e., PROLIFERATE: cross-scale components and insights from the transdisciplinary group)	Using mixed methods to locate, track, and respond to complex adaptive interaction, interconnections, and links between the sectors (adopters, users, and end-users) and the processes and stages of KT, given the person's sociodemographic characteristics and evaluated constructs in time/frequencies/occurrences (i.e., PROLIFERATE: cross-scale components and insights from the transdisciplinary group)	Using dialectical pluralism for co-designing adaptable mixed-method approaches/solutions *via* transdisciplinary and participatory action research methods that triangulate findings from: 1. the PROLIFERATE score and the cross-scale components; 2. the optimization construct (#5; and 3. the insights from the transdisciplinary group

**Table 4:** Terms defined according to the emerging adaptable framework: PROLIFERATE.

Term	Explanation
Big data (47)	Digital data (a considerable amount) captured *via* technological devices that require processing using computational or algorithmic procedures to draw responses to diverse research questions
Bayesian statistics and prediction modeling	Bayesian techniques are based on mathematical statistics to test and offer inferences about a matter of interest *via* Bayes’ theorem (48). In such a theorem, investigators update the probability of a hypothesis (*prior distribution*) by taking more evidence into its assessment (*posterior distribution*) (48). This Bayesian approach is fundamental to informing decision-makers. The method is used in medicine, quantum physics, biology, and the investment industries because of its prediction modeling capacities: estimating probability distributions of potential outcomes and allowing for random variation in inputs (i.e., stochastic changes) concerning the matters of interest (30, 48)
De-implementation (49)	A procedure of identifying and removing or substituting unsafe, irrelevant, and/or low-value practices, technologies, and/or processes (partially or entirely) *via* their empirical and evidenced-based evaluation; this includes developing unlearning methods to support and sustain the required behavioral, procedural, social, and/or contextual change
Falsifiability (50)	The condition of acknowledging falsification (e.g., disconfirmability or refutability): the logical possibility that a hypothesis, assertion, or theory can be revealed to be false through observation or an experiment (a test)
Transdisciplinary (24–27, 51)	The incorporation of knowledge—coming from different cultures, values, capabilities, and rationalities—from and with diverse stakeholders (experts and/or users) with interests to produce solutions that transcend the boundaries of their various fields and personal experiences
Net Promoter Score (52)	A method to evaluate and track the customer-centric value of products and/or services across large samples in a quantitative and replicable manner. It calculates the number of respondents expressing positive views about a product or service (“Promoters”), minus those with opposing views (“Detractors”), ignoring neutral responses (“Passive”)

The comparison of approaches in Table 3 presents the NPT level of complexity (45, 46) mostly around the social logic; the 2 × 2 conceptual map of influence on behaviors (42) as a tool consistent with the mechanistic logic; and PROLIFERATE as a multi-logic evaluation approach that can help prioritize and make sense of the elements of importance for stakeholders per their positioning within KT-cnm sectors and processes (24, 28–30). The embracement of PROLIFERATE toward different types of logic is observed in Table 3, as each descending row does not prevent predecessors’ approaches from occurring and being used in subsequent rows. In this way, the last row location of PROLIFERATE implies that it may, in a non-exclusive manner, absorb and mix the strategies of other methods in adaptable ways. Further, we provide snapshots of ongoing research in different healthcare settings within Australia to demonstrate how PROLIFERATE is being used while embracing different methodologies to evaluate various innovations.

## Results

To introduce the results, we return to Figure 1 and PROLIFERATE's multi-logic approach because its mixture of logics may result in several concepts and terms being interpreted differently depending on the reader's background. To facilitate a common language across logics within this manuscript, we created a glossary of critical terms in Table 4 to unify understandings around some of the ideas presented in the background and explored in the coming case exemplars.

To demonstrate PROLIFERATE's adaptability, in Table 5, we display snapshots of ongoing research in different healthcare settings within Australia; it exemplifies how PROLIFERATE is utilized within:

1. A community-based service: the innovation implemented is an interprofessional learning procedure within an allied health service (Health2GO). In this work, the transdisciplinary group (*n* = 96, across several sessions) co-designed the interprofessional learning procedure during focus groups that involved researchers, students, and teaching specialists from hearing, speech pathology, physiotherapy, vision, and health research areas (43).
2. A tertiary care service: the innovation is an Artificial Intelligence (AI) driven technology (RAPIDx_AI), which is implemented/tested *via* a randomized controlled trial (RCT), in which PROLIFERATE is evaluating its end-users’ feedback and integration within hospital workflows (24, 28, 30, 53). This adaptation of PROLIFERATE involves the creation of a transdisciplinary group (*n* = 15) to test the integration of the AI tool within hospital emergency departments. The group comprises experts in Bayesian models and statistical analyses; ethical and legal considerations; KT-cnm; medicine; RCTs; co-design; project management; cognitive sciences; behavior and health research; experimental design and big data; evaluation methods; science communication, health promotion, and marketing science; digital technologies and artificial intelligence; community representation and advocacy; non-profit organizations; psychology; social sciences and art; and nursing and clinical practice (24, 28, 30, 53).

**Table 5:** A snapshot comparison of two PROLIFERATE adaptations.

PROLIFERATE adaptations	Background	Objective	Methods	Findings/results	Conclusion
Health2Go study called: “Un-siloing allied health practice and interprofessional learning” (43)	An interprofessional service involves multiple professionals/caregivers collaborating to deliver quality care and comprehensive health services to clients. Leveraging the combined skills and perspectives in collaborative care improves client outcomes, saves time, and facilitates managing and coordinating chronic conditions. However, learning to coordinate different care stakeholders to manage multiple healthcare issues requires shifting power structures and embracing diversity to teach and apply such procedures	To co-design and evaluate interprofessional learning within a student-led clinic that offers optometry, audiology, physiotherapy, speech pathology, exercise physiology, occupational therapy, and nursing services to a diverse population (>50% born overseas). We intended to identify the challenges/opportunities to improve the sustainability of quality care *via* interprofessional learning	The interprofessional co-creation involved three co-design sessions, *n* = 32 people each, and a posterior sampling procedure. Ethics approval (No. 1858) and consent were received to apply: PROLIFERATE (Figure 2). After an initial implementation phase, the transdisciplinary group agreed on testing PROLIFERATE constructs using Net Promoting Score principles (see 52) *via* an anonymized online survey. Such principles measured the innovation's promoters, passive, and detractors of “Interprofessional learning (IL).” The passing or success of each construct (i.e., benchmark) was established considering the percentage of promoters in constructs #1, #2, and #4 (or the responses against detractors concerning construct #3). Construct #5 was assessed qualitatively by interpreting open answers to optimization questions	The results of the co-design sessions facilitated the implementation of IL *via* clinical learning activities outside the participant's discipline through observation and follow-up discussions. The online evaluation survey captured 15 survey respondents in the areas of hearing (20%), speech pathology (27%), physiotherapy (13%), vision (20%), and other (20%). Each PROLIFERATE construct (Figure 3) unveiled: 1. comprehension: promoters (47%), passive (40%), detractors (13%); 2. emotional responses: promoters (53%), passive (33%), detractors (13%); 3. barriers: responses provided against detractors (80%), promoters (13%), passive (7%); 4. motivation: promoters (40%), passive (47%), detractors (13%); and 5. Optimization: people requested targeted time or better scheduling and space to focus on IL	The design and implementation received a PROLIFERATE score of “Good Impact” and captured essential planning strategies for overcoming the motivation barriers around IL in allied health practices. Further investigation should incorporate the optimization suggestions and construct results in light of sociodemographics to longitudinally test IL using a bigger sample. This study brings evidence and methods for supporting the WHO's recommendation on developing a “collaborative practice-ready” health workforce that embraces differences around expertise and personal characteristics (culture, ethnicity, age, etc.) to improve healthcare
RAPIDx_AI (24, 28, 30, 53): a computer simulation study evaluating (predicting) the impact of AI implementation. Study called: “Predicting the implementation impact of RAPIDx_AI in South Australian (SA) emergency departments” (54)	There were 75,900 presentations to Australian public hospital emergency departments (EDs), with a principal diagnosis of coronary heart disease in 2020–2021. RAPIDx_AI is tested within a randomized controlled trial to test whether computer algorithms in hospital EDs can help doctors provide better care for patients by receiving guidance about diagnosing and treating patients with symptoms that may be due to their heart	To test a PROLIFERATE adaptation for predicting and measuring stakeholders’ perspectives about the implementation impact of RAPIDx_AI. This methodological adaptation was necessary because person-centered healthcare services require effective technology integration within clinical workflows to provide better patient care while considering the needs of all end-users involved and affected by AI or similar tech/practices/service changes	We introduced PROLIFERATE and incorporated Bayesian statistics as our data analysis method to benchmark the constructs. We created a protocol for this adaptation and modeled data (i.e., produced computer-simulated results) to demonstrate the evaluation and prediction capabilities of the method concerning the impact of RAPIDx_AI in simulated clinicians and communities (patients and their families). PROLIFERATE constructs were benchmarked at a 50% prediction concerning the 95% probability prediction intervals and credible intervals (2.50–97.50)—PROLIFERATE AI algorithm and data analysis were produced in R (software), *n* = 60 simulated scenarios	Our methodological innovation (protocol) is informed by 95% probability prediction and credible intervals on these domains of stakeholders’ perspectives: Comprehension, Emotional response; Uptake barriers; Motivation, and Optimization. Computer-simulated responses to a PROLIFERATE online survey predicted (Table 6) an Average Impact for RAPIDx_AI. The simulation results imply that the transdisciplinary group must implement motivational and emotional knowledge translation strategies for clinicians and the community to improve the perceived impact of RAPIDx_AI and its sustainability. Ethical approval for using PROLIFERATE (non-simulated evaluation) in 12 SA hospitals was granted by the Southern Adelaide Human Research Ethics Committee (SACHREC) (OfR no.272.20)	This AI adaptation of PROLIFERATE considers the non-linear characteristics of complex and adaptive workflows of acute care environments from an end-user perspective; it can monitor real-world clinical settings, research outcomes, and technological products by assessing their fitness *via* person-centered parameters and a co-designed transdisciplinary approach, which will be used to test and evaluate the RAPIDx_AI integration within clinical workflows to provide better patient care in SA

The Snapshots’ comparison of two PROLIFERATE adaptations (Table 5) exemplifies *what* is required and/or *how* PROLIFERATE methodological adaptations are becoming fit for purpose within two different innovations and objectives. For instance, in adaptation 1 (Health2Go), the evaluation detected a lack of motivation leading to a score of “good impact” because of more passive than promoters’ responses in construct #4 (motivation to change); to address this issue, data from construct #5 (optimization) informed the transdisciplinary group about potential ways to change this situation: offering stakeholders insight, according to each type of learners and their interactions with others; developing solutions targeting better times for interprofessional learning; delivering better schedules; and providing space to focus on the process (43).

In RAPIDx_AI, the transdisciplinary group pre-established a success benchmark of 50% for each end-user type (i.e., clinicians and community, as per Table 6). The idea of this simulation is to demonstrate that based on that information (big data approach), the transdisciplinary group can co-design KT activities, interventions, and solutions to move constructs above the benchmark for clinicians and the community concerning the undesirable predictions (in the lower level of the credible interval) about the motivations and emotions concerning RAPIDx_AI potential impact (see Table 1) (24, 28, 30, 53).

**Table 6:** The predictive result from RAPIDx_AI modeling via Bayesian statistics in R (24, 28, 30, 53).

Intervention Group	Prior	Mean	0.025	0.975
**Clinicians**	Uptake barriers	0.86	0.68	0.97
	Comprehension	0.81	0.60	0.95
	Emotion	0.60	0.40	0.79
	Motivation	0.66	0.44	0.85
	Optimization	0.76	0.56	0.91
**Community**	Uptake barriers	0.86	0.70	0.97
	Comprehension	0.81	0.62	0.94
	Emotion	0.62	0.40	0.81
	Motivation	0.66	0.44	0.82
	Optimization	0.77	0.56	0.93

The prediction of RAPIDx_AI's impact unveiled this PROLIFERATE score: “average impact”; because it found only two successful constructs (over the 50% benchmark, including the credibility intervals): comprehension—construct #1—and uptake barriers—construct #3, and identified the other two (motivation—construct #4, and emotion—construct #2) below the agreed benchmark (Table 6). Qualitative data analysis from the assessment of construct #5 (optimization) must be considered to create KT strategies around the constructs, according to the sociodemographic variables and the identified KT-cnm sectors and stages concerning clinicians and community members (24, 28, 30, 53).

These examples of using the PROLIFERATE scoring system are collected in both studies *via* an online survey to investigate the triangulation behind the PROLIFERATE cross-scale components (as described in Figures 4–6). In both case exemplars (Health2Go and RAPIDx_AI), each sector's sociodemographic characteristics and responses to construct #5 (optimization) should have been considered against the transdisciplinary group's insights; such triangulation would have determined the best KT approach that addresses cross-scale findings. However, these examples reflect incipient studies that need more progress to share such experiences.

A takeaway from the current status of PROLIFERATE is that within the case examples presented, the transdisciplinary team cross-pollinated ideas based on their experiential learning, aiming to acquire, utilize, or master individual and/or collective skills and capabilities for collaborative research (51) (Box 1).

Box 1 **These capabilities underpinned core values that were explicitly put forward and explored with each PROLIFERATE adaptation (see co-design evaluation procedure and Figure 2).**

• to undertake research that crosses disciplinary boundaries (51);
• to develop and apply tools and frameworks in new situations (51);
• to sustain an appreciation for the importance of the particular or granular aspects of problems (51);
• to utilize and understand pluralism (51);
• to acknowledge and communicate complexity effectively (51);
• to understand and utilize reflexivity (51);
• to actively empower collective leadership centered around the core values while navigating the power dynamics (51);
• to reimagine and work toward sustaining research livelihood (51);
• to manage/work with and for a research team beyond institutional boundaries and projects (51);
• to establish trust in collaboration (51);
• to be egalitarian (51);
• to be humble (51); and
• to build societal capacity for democratic struggle (51).

Embarking on this journey to inform the nature and body of work requires commitment and support, alongside investment of time and effort—most often to absorb the backlash due to power dynamics and deeply entrenched “resistance to change.” The development of the PROLIFERATE framework tried to bring a conglomerate of knowledge and wisdom (like a snowball) collectively by collaborating and undertaking research projects within the domain of applied KT, IS, and health systems research. This shared experience (history or collaboration projects) is enriching, despite differing views, methods, cultures, or perspectives. However, it implies that all participants, co-authors, and partakers have a vision that is based on the listed core values, so they all gain something relevant by reaching toward it. Most studies of this transdisciplinary nature refer to the high cost behind such collaborative activities, mainly referring to involving non-academic peers; we believed that their budgeted and supported involvement is an ethical imperative that must always be part of any multistakeholder design (14, 15, 19).

Those interested in applying an adaptation of the PROLIFERATE framework to their programs, projects, products, or procedures must consider the framework flexibility; this means their investments (cost, skills, and time) would depend on the context and matter to be evaluated while forming a transdisciplinary group, fomenting the core values, so that they agreed on goals, and benchmarking methods. This process could be articulated into a straightforward and rapid project or could become a prolonged sustainability cycle; from our point of view, it promises returns on investment because it measures and can optimize innovations and solutions considering their context complexity while delivering adaptive strategies. This perspective comes from our own longitudinal and transdisciplinary co-design journey concerning these evaluation endeavors. They have been powered and moved by long-term aims that feed into one or several research programs. This process requires a longstanding plan, including envisioning, thought leadership, and appropriate investments. We have benefited from the ingenuity of articulating different projects into a programmatic and impactful vision in which all part-takers gain something in the long run.

To illustrate the navigation process and the associated complexities of our long-term evaluation design, we mapped the networks of collaboration from which the PROLIFERATE framework is emerging. Usually, a more extensive view of the network exists in real-world scenarios as it involves more than one investigator and stakeholders representing several institutions and groups. However, in this paper, for simplification purposes, we presented the lead author’s (investigator's) network because it gives us a sense of the least we can capture empirically (as best as we can with the current measurement tools) through her published works (from the period 2019 until the present, 2023). This network represents the internal and external stakeholders (academic and non-academic; health and non-health; practice and policy), influencing implicitly, explicitly, directly, or indirectly the development of PROLIFERATE and its emerging iterations or adaptations.

**Figure 7:**
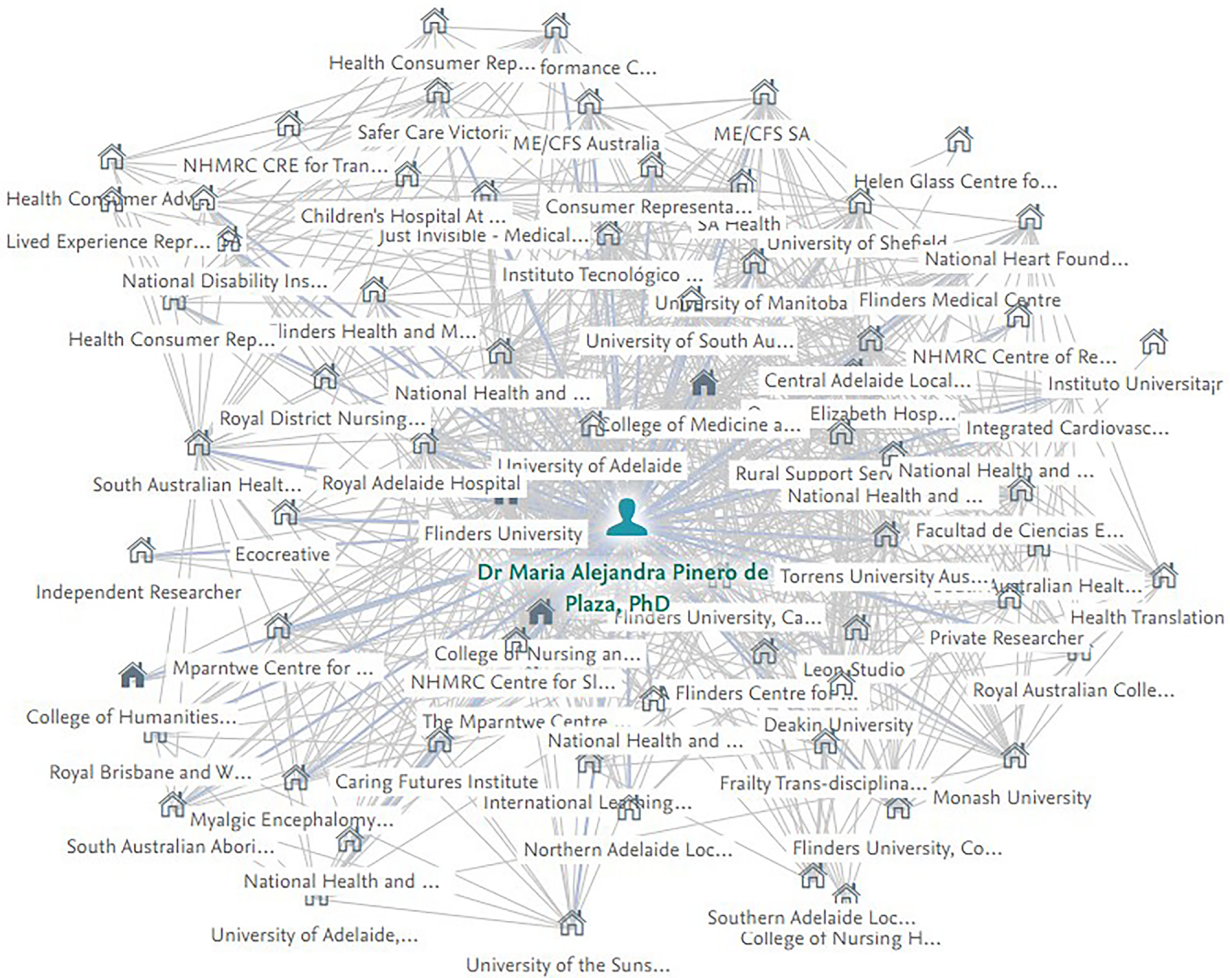
Lead author emerging PROLIFERATE network—developing and maturing over time (2019–2023). Data are based on the published work and co-authorship networks generated by the “ResearchNow” open online platform of Flinders University.

Figure 7 presents a PROLIFERATE co-creation network as a growing connectivity structure empowered with similar core values and the long-term goals underpinning KT and IS approaches. For example, generalizing some of our experiences around transdisciplinary goals, researchers on this network wanted to co-create the framework because of their investigative and academic interest in co-design and translation in a real-world setting. Health consumers wanted to influence healthcare services and research procedures and make their voices heard and influential within the discovery and implementation processes. Clinicians needed to demonstrate the effect and impact of their interventions to improve care and attract and justify funding. Artists wanted to demonstrate how their methods could generate an impact and social change, and industry wanted to be backed up by evidence-based research. This win-win scenario for the network does not end after achieving a single objective or a specific endpoint but intends to continue in the journey while learning its lessons. Each stakeholder or person collaborating and participating in any adaptation of PROLIFERATE or its associated KT studies is willingly a part of a transdisciplinary program to which they bring their own networks, knowledge, and agendas/interests. In this democratic process, they seem to organically (and eventually, after induction, intentionally) recognize the intersecting spaces (per the KT-cnm) in which synergy and dialectical relationships seem beneficial strategies to attain, maintain, or gain their respective long-term goal.

## Discussion

PROLIFERATE allows and promotes the utilization of metrics (e.g., measurable strategies such as data science) to help the transdisciplinary group falsify or test their assumptions about the dynamics of the health systems and the stakeholders they represent (17, 43, 44, 55). PROLIFERATE's adaptable nature and its transferable scoring system can be used to compare and measure by how much of a difference an innovation or a solution has impacted; this ability extends to predictive models of such impacts (24, 28, 30, 53). However, further iterations and longitudinal analyses must elucidate PROLIFERATE's utility and relevance across time and with bigger sample sizes. Its emerging status demonstrates the method's applicability and flexibility. However, the case examples are still in progress and not mature enough to:

1. illustrate the PROLIFERATE process in the long run, its obstacles, benefits, or the effects of the final steps of its procedure concerning implementing the strategies recommended by the transdisciplinary group and their impact; and
2. map or describe the cross-scale components that reflect the structural patterns of multiple interactions and connections of multilevel social networks to facilitate or limit the uptake, spread, and scale-up of new knowledge in the form of innovations and/or solutions and change.

A call to utilize and test PROLIFERATE is extended so that peers can evaluate its advantages and limitations within other healthcare services, products, procedures, and challenges. Such iterations may decant and percolate the dialectical pluralist approach and the multi-logic attributes enabling sustainable change or obstructing it. Peers could test the re-orientation of networks to facilitate the implementation of change by promoting effective connectivity between the five KT-cnm processes; this can be explored in future research by introducing tools such as the 2 × 2 conceptual map of influence on behaviors (42) and its mapping capabilities. They may enhance the recommendations and strategies of the transdisciplinary group by tailoring the KT-cnm structures *via* influencing conscious or unconscious behaviors (42).

A challenge around agreeing on implementing optimization strategies can emerge despite the dialectical pluralist approach. Even when inclusiveness should guide PROLIFERATE's co-design work, the transdisciplinary group can be seen as a miniature representation of the whole health system. Therefore, to diminish ideological and many other differences, the group's attention to cross-scale findings must be their focus to inform decisions and recommendations (an evidence-based emphasis) (31, 38–40). Yet, the difference between members’ agendas is expected. Therefore, other avenues may point to research projects adapting PROLIFERATE to gaming frameworks, such as the Octalysis Framework. This could help direct behaviors, as done with game players, toward certain activities or decisions (56). Such a combination could help the transdisciplinary group testing if behavioral drivers that move game players can influence and benefit behavioral change and KT and IS. For example, the first driver of the Octalysis Framework is called “epic, meaning and calling”; it involves activities in which the person's motivation is acting safely and responsibly for a cause greater than themselves (56). These activities may induce change from not-for-profit stakeholders associated with a particular innovation.

In contrast, the fifth driver of the Octalysis Framework—“social influence and relatedness”—incorporates social elements that motivate the person to function *via* mentorship, social acceptance, and considering other influences such as competition and envy (56). This driver may influence behavioral change in health practitioners, industry sectors, or academics. Similar methods around stimulating drivers, triggers, and motivators of behavior have been used by members of the transdisciplinary group that created PROLIFERATE; they were applied in marketing studies to identify buyers and users of luxury items (57) and in health promotion to identify patterns of healthy and unhealthy dietary habits (55). Consequently, future iterations and adaptation of the PROLIFERATE evaluation framework could allow testing such techniques and their abilities to improve the co-designing, measuring, and optimizing of innovations and solutions within complex adaptive health systems.

## Conclusion

An essential requirement to face today's health challenges is taking a complex view of the impacts or effects of solutions and innovation within the health systems. Such approaches need further research around multi-logic methods because they invite crossing traditional scientific boundaries to bring new ways of understanding our human physical, biological, ecological, and social dimensions (17, 43, 44, 44, 58). Consistently, we share PROLIFERATE as one of the first frameworks operationalizing the KT-cnm. This operationalization adds a novel perspective to the individual's agency in the system by considering their responses to innovations, including tech-enabled solutions within different healthcare settings. This work provides structured co-design and co-facilitation processes that help engage multiple stakeholders in dynamic and productive ways by measuring and optimizing behavioral patterns around innovation, considering the complexities of their uptake, spread, and scale-up.

## Data Availability

The original contributions presented in the study are included in the article, further inquiries can be directed to the corresponding author.

## References

[1] Sheikh K Abimbola S, World Health Organization. Learning health systems: pathways to progress: flagship report of the alliance for health policy and systems research (2021).

[2] Friel S Marmot M McMichael AJ Kjellstrom T Vågerö D. Global health equity and climate stabilisation: a common agenda. Lancet. (2008) 372(9650):1677–83. 10.1016/S0140-6736(08)61692-X 18994666

[3] Pinero de Plaza MA Beleigoli A Mudd A Tieu M McMillan P Lawless M Not well enough to attend appointments: telehealth versus health marginalisation. In: IOS Press Series e-Book: Studies in health technology and informatics. IOS PRESS. (2021). p. 72–9.

[4] Fullman N Barber RM Abajobir AA Abate KH Abbafati C Abbas KM Measuring progress and projecting attainment on the basis of past trends of the health-related sustainable development goals in 188 countries: an analysis from the global burden of disease study 2016. Lancet. (2017) 390(10100):1423–59. 10.1016/S0140-6736(17)32336-X 28916366 PMC5603800

[5] Ewert B Wallenburg I Winblad U Bal R. Any lessons to learn? Pathways and impasses towards health system resilience in post-pandemic times. Health Econ Policy Law. (2022):1–16. 10.1017/S1744133122000238 36121039

[6] Pinero de Plaza MA Beleigoli A Brown S Bulto LN Gebremichael LG Nesbitt K Effectiveness of telehealth versus standard care on health care utilization, health-related quality of life, and well-being in homebound populations: a systematic review protocol. JBI Evid Synth. (2022) 20(11):2734–42. 10.11124/JBIES-21-00410 35975313

[7] Pinero de Plaza MA Brown S Wu C-J Robyn C McBride K Gebremichael L System enablers and barriers to continuity of care for First Nations people living with chronic conditions: A rapid qualitative review protocol: figshare (2022). Available at: https://figshare.com/articles/online_resource/System_enablers_and_barriers_to_continuity_of_care_for_First_Nations_people_living_with_chronic_conditions_A_rapid_qualitative_review_protocol/20310117 and https://ndownloader.figshare.com/files/36328944. (Accessed August 9, 2022)

[8] Mudd A Feo R Pinero de Plaza MA Tieu M Paia SY The use of digital technologies in the inpatient setting to promote communication during the early stage of an infectious disease outbreak: a scoping review. Telemed e-Health. (2022). 10.1089/tmj.2021.0615 35758765

[9] Haakenstad A Yearwood JA Fullman N Bintz C Bienhoff K Weaver MR Assessing performance of the healthcare access and quality index, overall and by select age groups, for 204 countries and territories, 1990–2019: a systematic analysis from the global burden of disease study 2019. Lancet Glob Health. (2022) 10(12):e1715–43. 10.1016/S2214-109X(22)00429-6 36209761 PMC9666426

[10] Morel CM Acharya T Broun D Dangi A Elias C Ganguly N Health innovation networks to help developing countries address neglected diseases. Science. (2005) 309(5733):401–4. 10.1126/science.1115538 16020723

[11] Beleigoli A Nicholls SJ Brown A Chew DP Beltrame J Maeder A Implementation and prospective evaluation of the Country Heart Attack Prevention model of care to improve attendance and completion of cardiac rehabilitation for patients with cardiovascular diseases living in rural Australia: a study protocol. BMJ Open. (2022) 12(2):e054558. 10.1136/bmjopen-2021-054558 35173003 PMC8852732

[12] Deponte D Fossa G Gorrini A. Shaping space for ever-changing mobility. COVID-19 lesson learned from Milan and its region. TEMA. (2020):133–49. 10.6092/1970-9870/6857

[13] Alam F Almaghthawi A Katib I Albeshri A Mehmood R. iResponse: an AI and IoT-enabled framework for autonomous COVID-19 pandemic management. Sustainability. (2021) 13(7):3797. 10.3390/su13073797

[14] Daepp MI Binet A Gavin V Arcaya MC, The Healthy Neighborhoods Research Consortium. The moving mapper: participatory action research with big data. J Am Plann Assoc. (2022) 88(2):179–91. 10.1080/01944363.2021.1957704

[15] Wiles LK Kay D Luker JA Worley A Austin J Ball A Consumer engagement in health care policy, research and services: a systematic review and meta-analysis of methods and effects. PLoS One. (2022) 17(1):e0261808. 10.1371/journal.pone.0261808 35085276 PMC8794088

[16] Nabyonga-Orem J Asamani JA. Ensuring the right to health along the life course. Lancet Glob Health. (2022) 10(12):e1689–90. 10.1016/S2214-109X(22)00458-2 36400071 PMC9681659

[17] Zhao J. Is there such a thing as a symptom cluster: the paradigm shift in symptom science requires a philosophical reflection. Asia-Pac J Oncol Nurs. (2022) 9(5). 10.1016/j.apjon.2022.03.008 PMC9130072 35633916

[18] Arcaya M Raker EJ Waters MC. The social consequences of disasters: individual and community change. Annu Rev Sociol. (2020) 46:671–91. 10.1146/annurev-soc-121919-054827

[19] Slattery P Saeri AK Bragge P. Research co-design in health: a rapid overview of reviews. Health Res Policy Syst. (2020) 18(1):1–13. 10.1186/s12961-020-0528-9 32046728 PMC7014755

[20] Greenhalgh T Robert G Macfarlane F Bate P Kyriakidou O. Diffusion of innovations in service organizations: systematic review and recommendations. Milbank Q. (2004) 82(4):581–629. 10.1111/j.0887-378X.2004.00325.x 15595944 PMC2690184

[21] Nilsen P. Making sense of implementation theories, models, and frameworks. Springer International Publishing (2020). p. 53–79. 10.1186/s13012-015-0242-0 PMC4406164 25895742

[22] Greenhalgh T Papoutsi C. Spreading and scaling up innovation and improvement. Br Med J. (2019) 365. 10.1136/bmj.l2068 PMC6519511 31076440

[23] Archibald M Ambagtsheer R Lawless MT Thompson MO Shultz T Chehade MJ Co-designing evidence-based videos in health care: a case exemplar of developing creative knowledge translation “evidence-experience” resources. Int J Qual Methods. (2021) 20:16094069211019623.

[24] Pinero de Plaza MA. PROLIFERATE: An adaptable framework with tools to evaluate different processes, outputs, and products via participatory research: figshare (2022) Available at: https://figshare.com/articles/online_resource/PROLIFERATE_An_adaptable_framework_with_tools_to_evaluate_different_processes_outputs_and_products_via_participatory_research/20374005. (Accessed July 29, 2022)

[25] Pinero de Plaza MA. A transdisciplinary research program addressing complex health research problems. JBI Evid Implement Bull. (2022) 30(1).

[26] King G Strachan D Tucker M Duwyn B Desserud S Shillington M. The application of a transdisciplinary model for early intervention services. Infants Young Child. (2009) 22(3):211–23. 10.1097/IYC.0b013e3181abe1c3

[27] Aslan D. Can transdisciplinary approaches contribute to the COVID-19 fight? Glob Health Promot. (2021) 28(2):72–7. 10.1177/17579759211002376 33908313

[28] New ways to solve complex problems and PROLIFERATE. Flinders University (2022). Available at: https://open.flinders.edu.au/articles/data_management_plan/New_Ways_to_Solve_Complex_Problems_and_PROLIFERATE/21365796.

[29] Pinero de Plaza M Archibald M Lawless M Ambagtsheer R Mudd M McMillan P PROLIFERATE: an adaptable framework to evaluate participatory research products. Preprint (Version 2). Available at: https://www researchs quare com/article/rs-146129/v2. 2021. (Accessed November 22, 2022)

[30] Pinero de Plaza MA Lambrakis K Morton E Beleigoli A Lawless M McMillan P PROLIFERATE: a tool to measure impact and usability of AI-powered technologies. Digital Health Institute Summit. Melbourne: The Australasian Institute of Digital Health Summit (2022). Available at: https://researchnow.flinders.edu.au/en/publications/proliferate-a-tool-to-measure-impact-and-usability-of-ai-powered. (Accessed March 13, 2022)

[31] Kitson A Brook A Harvey G Jordan Z Marshall R O’Shea R Using complexity and network concepts to inform healthcare knowledge translation. Int J Health Policy Manag. (2018) 7(3):231. 10.15171/ijhpm.2017.79 29524952 PMC5890068

[32] Scotland ES. Evaluation methods and tools—resources—Body Map: Evaluation Support Scotland. Available at: http://www.evaluationsupportscotland.org.uk/media/uploads/resources/pdf_method_-_body_map.pdf. (Accessed March 14, 2021)

[33] Jaatun EAA Fallon M Kofod-Petersen A Halvorsen K Haugen DF. Users’ perceptions on digital visualization of neuropathic cancer-related pain. Health Informatics J. (2019) 25(3):683–700. 10.1177/1460458217720392 28747078

[34] Barr A Dailly J. LEAP: A Manual for Learning Evaluation and Planning in Community Development (2007).

[35] Orme J Powell J Taylor P. Public health for the 21st century. McGraw-Hill Education (2007).

[36] Johnson RB. Dialectical pluralism: a metaparadigm whose time has come. J Mix Methods Res. (2017) 11(2):156–73. 10.1177/1558689815607692

[37] Nilsen P Thor J Bender M Leeman J Andersson-Gäre B Sevdalis N. Bridging the silos: a comparative analysis of implementation science and improvement science. Front Health Serv. (2022) 18. 10.3389/frhs.2021.817750 PMC10012801 36926490

[38] Conroy T Pinero de Plaza MA Mudd A Mitchell M Kitson A. Measuring fundamental care using complexity science: a descriptive case study of a methodological innovation. J Clin Nurs. (2021) 00, 1–10. 10.1111/jocn.15905 34137100

[39] Pinero De Plaza MA Conroy T Mudd A Kitson A. Using a complex network methodology to track, evaluate, and transform fundamental care. Stud Health Technol Inform. (2021) 284:31–5. 34920462 10.3233/SHTI210656

[40] Bergström J Sidney WA. Bridging the macro and the micro by considering the meso reflections on the fractal nature of resilience. Ecol Soc. (2014) 19(4). 10.5751/ES-06956-190422

[41] Wang P Robins G Pattison P Lazega E. Social selection models for multilevel networks. Soc Networks. (2016) 44:346–62. 10.1016/j.socnet.2014.12.003

[42] Nilsen P Potthoff S Birken SA. Conceptualising four categories of behaviours: implications for implementation strategies to achieve behaviour change. Front Health Serv. (2022) 1:795144. 10.3389/frhs.2021.795144 36926485 PMC10012728

[43] Pinero de Plaza MA Jacobs D Chipchase L. Un-siloing allied health practice and interprofessional learning: a co-design and evaluation case study. In: Pinero de Plaza MA Jacobs D Chipchase L, editors. The national health and medical research council (NHMRC) research translation long weekend 2022. Embracing Diversity. (2022).

[44] Pinero de Plaza MA Dafny H Lawless M McMillan P Buchanan R Leon Z Holding the suspension bridge of ongoing high-quality care. 55th Australian Association *o*f Gerontology (AAG) Conference: *The Future* of *Ageing Well* (2022).

[45] May CR Albers B Bracher M et al Translational framework for implementation evaluation and research: a normalisation process theory coding manual for qualitative research and instrument development. Implement Sci. (2022) 17(1):19. 10.1186/s13012-022-01191-x 35193611 PMC8861599

[46] May CR Cummings A Girling M et al Using normalization process theory in feasibility studies and process evaluations of complex healthcare interventions: a systematic review. Implement Sci. (2018) 13(1)80. 10.1186/s13012-018-0758-1 29879986 PMC5992634

[47] Favaretto M De Clercq E Schneble CO Elger BS. What is your definition of big data? Researchers’ understanding of the phenomenon of the decade. PLoS One. (2020) 15(2):e0228987. 10.1371/journal.pone.0228987 32097430 PMC7041862

[48] Lee MD Wagenmakers EJ. Bayesian cognitive modeling: A practical course. Cambridge University Press (2014).

[49] Upvall MJ Bourgault AM. De-implementation: a concept analysis. In: Upvall MJ Bourgault AM, editors. Nursing forum. Wiley Online Library (2018). 10.1111/nuf.12256 29691868

[50] Popper K. The logic of scientific discovery. Routledge (2005).

[51] O'Donovan C Michalec O Moon J. Capabilities for transdisciplinary research. An evaluation framework and lessons from the ESRC Nexus Network+ (August 5, 2020) SWPS 2020;12.

[52] Owen R. Net promoter score and its successful application. Mark Wisdom. (2019):17–29.

[53] Pinero de Plaza MA Lambrakis K Causil CJ Ramos FM Chew D Beleigoli A Predicting the implementation impact of RAPIDx AI in South Australian emergency departments. Australia: South Australian Cardiovascular Showcase (2022).

[54] HTSA. RAPIDx AI, Using Artificial Intelligence to Improve Emergency Care of People with Chest Pain Health Translation SA (2019) Available from: https://healthtranslationsa.org.au/projects/rapidx-ai/ (Accessed February 9, 2023)

[55] Pinero de Plaza MA Taghian M Marmolejo-Ramos F Barrera-Causil CJ Hall J. Investigating salience strategies to counteract obesity. Health Promot Int. (2021) 36(6):1539–53. 10.1093/heapro/daaa123 33599262

[56] Chou Y-k. Actionable gamification: beyond points, badges, and leaderboards. Packt Publishing Ltd. (2019).

[57] Loureiro SMC Pinero de Plaza MA Taghian M. The effect of benign and malicious envies on desire to buy luxury fashion items. J Retailing Consum Serv. (2020) 52:101688. 10.1016/j.jretconser.2018.10.005

[58] Tieu M Mudd A Conroy T Pinero de Plaza A Kitson A. The trouble with personhood and person-centred care. Nurs Philos. (2022) 23(3):e12381. 10.1111/nup.12381 35416420

